# Acceptability and Feasibility of Home-Based Human Papillomavirus Self-Testing as Primary Screening for Cervical Cancer Detection in the State of Alabama

**DOI:** 10.1007/s10900-026-01554-1

**Published:** 2026-03-06

**Authors:** Van Thi Ha Nghiem, Shelita Smith, James Shi, Alexandra McBrayer, Claudia Hardy, Akila Subramaniam, Kevin Fontaine, Renee Heffron, Andrzej Kulczycki

**Affiliations:** 1 University of Alabama at Birmingham School of Public Health, Birmingham, AL, USA; 2 University of Alabama at Birmingham Heersink School of Medicine, Birmingham, AL, USA; 3 Department of Health Policy and Organization, University of Alabama at Birmingham School of Public Health, 1665 University Blvd., Suite 320D, Birmingham, AL 35233, USA

**Keywords:** HPV, Self-testing, Cervical cancer, Alabama, Access to care

## Abstract

Alabama has the highest cervical cancer mortality and sixth highest incidence rates nationwide. We assessed the acceptability and feasibility of at-home HPV self-testing for cervical cancer screening in Alabama. Eligible women (ages 30–64y, no Pap last 3.5y, not being pregnant, no hysterectomy history, and satisfying either rural residency or being African American) were recruited by community health workers (CHW), foodbanks, churches, and trusted relationships in targeted communities. Participants received a mailed package including an Evalyn^®^ HPV self-test kit (for analysis against high-risk (HR) HPV-16, −18, and -other HR-groups); and a questionnaire seeking information on health literacy, healthcare experience and attitudes about the HPV self-testing. CHWs recruited 86% of participants. Thirty-five (60%) out of 58 consented-to-participate women returned the completed questionnaires and test kits. Half reported having public insurance, a quarter were uninsured. Half had > 5 sex partners. About 40–77% had incorrect answers to HPV knowledge questions. Ten participants (29%) reported having little trust in doctors. Over 90% of the self-collected samples were analyzable. Three samples were positive with HR-16 and −18 (received referral information for follow-up) but either negative or invalid with other HR-groups, and 29 tests were negative for all HR-groups. Almost all participants (94%) were comfortable with receiving test-kits via mail, and most (83%) preferred home- to clinic-based screening. Overall, HPV self-testing was acceptable among women recruited in community setting. Health literacy, medical trust, and the CHW role can be emphasized in future work to promote HPV self-testing to eliminate cervical cancer.

## Introduction

With recent improvements in care, cervical cancer has become less than half as lethal as it was in the 1970s [1]. However, cervical cancer still disproportionately affects poor and racial minority women. Among U.S. states, Alabama has the highest cervical cancer mortality rate (3.2/100,000) [2] and the sixth highest incidence rate [3]. Limited sexual health education, low human papillomavirus (HPV) vaccination rates, and a decline in the number of physicians and medical facilities, especially in rural areas, contribute to this disparity [4].

The most common cause of cervical cancer is HPV, which is usually transmitted through sexual activity [5]. African American women especially receive low-quality care that is often too late [6]. Alabama has a widespread shortage of medical professionals compounded by the refusal to expand Medicaid, pushing more rural hospitals to cut essential services to avoid closure [7]. The coverage of obstetricians and gynecologists has decreased significantly, with only 26 counties having hospitals that provided obstetric services in 2025, compared to 58 in 1980 [8]. Additional barriers to accessing cervical cancer care include economic hardship, the lack of insurance coverage, the need for long-distance travel to receive care due to hospital closures [9], limited awareness of free screening programs and the ending of Medicaid coverage after cancer remission. Around 2018, only 18% of eligible women were using the Alabama Breast and Cervical Early Detection Program—a free screening program for 40–65 year-old women without health insurance and household income < 250% of the federal poverty level [10, 11].

Currently, national guidelines recommend regular cervical cancer screening from ages 21 to 65 years with methods such as cytology only, co-testing (combining HPV test and Pap test), or primary HPV testing [12]. Primary HPV testing at home is a clinically effective screening tool used increasingly worldwide [13, 14]. In a study of Danish women, participation was significantly higher in women who received at-home kits or were invited to opt-in for at-home kits (mailed to home, self-testing, returned to analysis labs) compared to women who were invited to attend regular cytology screening at general practitioners’ clinics [15]. Women in Italy had high self-test kit return rates during the COVID pandemic [16, 17], making at-home tests an appealing screening option when in-person screening is unavailable [16, 17]. The mailing of at-home kits has also shown success in targeting underscreened women that are never screened or overdue for repeating the guideline-recommended screening [18]. Women who use self-test kits found them to be easy to use, with the most common reason for not using the kits being medial mistrust [19]. In mid-2024, the Food and Drug Administration (FDA) approved the self-collection approach for HPV testing as a primary cervical cancer screening method. The Human Resources and Services Administration guideline now endorses HPV testing as the preferred cervical cancer screening approach, either administered by the patient or the clinician [20]. Self-collection’s cost-effective [21] nature has the potential to significantly boost screening rates among women in Alabama and other states with high cervical cancer disease burdens by eliminating the barriers of financial hardships and lack of medical professionals. Previous studies (in both pre- and post-FDA approval eras) have examined the efficacy of this self-testing HPV method in multiple U.S. geographies including Washington [22], Texas [23], Appalachian states (Kentucky, Ohio, Virginia, and West Virginia) [24], and Florida [25]. An earlier study suggested that HPV self-collection could be acceptable and feasible to African American women in the Mississippi Delta [26], but this was conducted in a clinical setting but not in the community.

Women in Alabama face critical barriers preventing widespread screening for cervical cancer. To consider ways to close this gap, we aimed to examine acceptability of home-based HPV self-sampling for underserved and underscreened women in Alabama. Findings from our study would be helpful for planning future work to prevent and manage cervical cancer in the state of Alabama and elsewhere. Particularly, findings may inform public health programs such as Operation WIPE OUT, the state initiative to eliminate cervical cancer as a public health problem in Alabama by improving awareness about HPV and cervical cancer prevention as well as promoting timely access to preventive and therapeutic services [27].

## Methods

### Study Design

We conducted a cross-sectional pilot study to examine the acceptability and feasibility of home-based HPV testing for cervical cancer screening among women recruited from Alabamian community settings. The PRECEDE-PROCEED framework, a commonly used framework for disease screening in community settings [28], was used to guide the research design. Key model constructs include predisposing, reinforcing, and enabling factors that shed light into facilitators and barriers of using HPV self-testing in our specific underserved women [29].

### Participants

The study recruited underserved women [30] living in Alabama who were overdue for cervical cancer screening. Eligible participants also had to either reside in rural areas [31] or be African American [9, 32], be 30–64 years old; have a cervix and not be pregnant. Additionally, they should not have had a Pap test for at least three and a half years, which goes beyond the every-three-year interval for repeated screening recommended by the American Cancer Society Guidelines for the Prevention and Early Detection of Cervical Cancer [33].

### Recruitment

We leveraged multiple recruitment strategies including in-person locations, mobile clinics, and organization meetings and listservs. We distributed study brochures at local foodbanks in the peripheries of Birmingham and Tuscaloosa urban areas of central Alabama. Study team members reached out to engage with leaders of these foodbanks, local churches and a local obstetric/gynecologic clinic for recruitment. To this end, we also left our study brochures at their premises and had some of these leaders promote our study via word of mouth.

The study team additionally recruited participants in rural southern Alabama by leveraging a relationship with experienced community coordinators in Butler, Macon, and Bullock counties of Alabama. They were also community health workers and members of our institution’s partnership for community engagement. This approach made use of a long-standing model providing cancer education and community engaged research in medically underserved communities. In addition, study flyers, including a designated informational webpage (including study phone number and email address) [34], were sent to our institution-led mobile bus which provided several preventive cardiovascular services to select underserved areas in Alabama for opportunities to recruit eligible participants. Study information was also broadcast via regular meetings and listservs of the Alabama Comprehensive Cancer Control Coalition—a statewide network of physicians, patients and survivors, community-based organizations, and state-based agencies collaborating to reduce cancer burden in the state. Women interested in our study would contact the research team by phone or email and receive more information about participation.

### Procedure

**Fig. 1:**
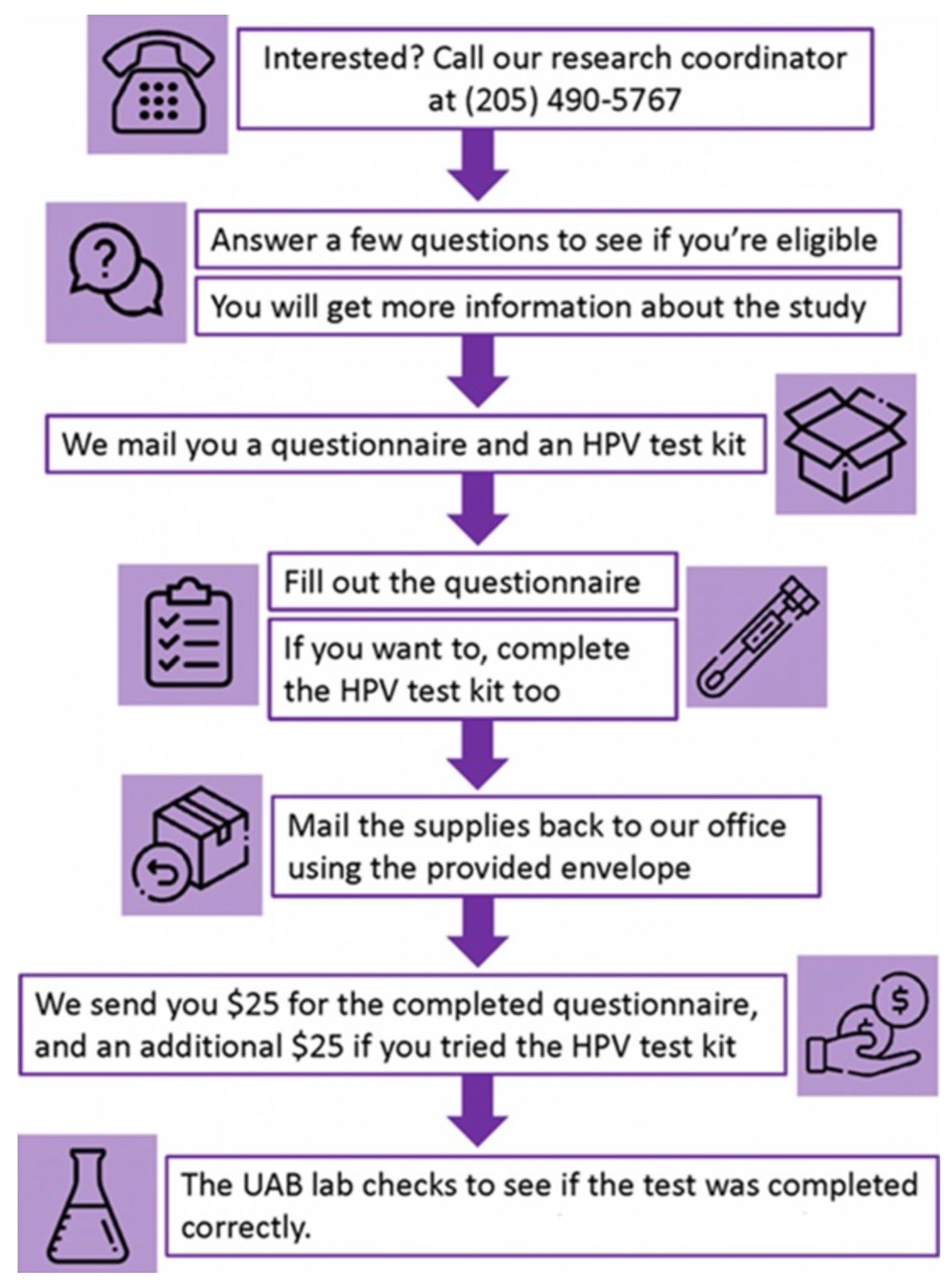
Study procedure flowchart. Procedure to enroll eligible participants and to follow up with them throughout the study questionnaire and sample collection steps

Women interested in the study were contacted by phone and screened for eligibility. Eligible women were consented over the phone and mailed a study package that included a paper questionnaire, an Evalyn^®^ Brush HPV self-test kit (Rovers^®^ Medical Devices, Oss, Netherlands) and the manufacturer’s instructional manual, a prepaid return envelope, a printed informed consent form (for the purpose of documentation at the participant’s end), a study information flyer, and an instruction sheet on how to reach out to the study team in the event of questions or problems. Partway through recruitment, we developed an IRB-approved letter to help share results with participants. This development was based on multiple participants’ persistent interest in having access to their results. Those who wanted to receive the testing result would receive a letter notifying them of their results and, if applicable, would be referred for follow-up with gynecologic clinics and the Alabama Early Breast and Cervical cancer screening program. The instruction sheet emphasized that testing was conducted for research purposes and was not a firm clinical diagnosis. Participants were compensated for their time with a $25 gift card for completing the questionnaire and another $25 gift card for completing the self-test kit (Fig. 1).

### Specimen Collection and Processing

The Evalyn^®^ brush was used for self-collection of cervical/vaginal samples. The women followed the written instructions on the package, proceeded the self-collection and sent it back to our research office. We used the Cobas^®^ 4800 HPV test system (Rocher Molecular Systems, Inc., Basel, Switzerland) and the ThinPrep^®^ solution (Hologic, Inc., Marlborough, MA) to analyze the samples. The laboratory process was validated using two external HPV samples that were previously tested by our institution’s Ob-Gyn research and diagnostic laboratory in accordance to standard protocols [35, 36]. We used carrier tracking services and documented dates to track time duration for the samples being in transit and waiting for laboratory analysis. Outdoor temperature during samples being in transit was also documented to assess, if necessary, the possible impact of this temperature on the laboratory-analysis-related performance of the self-collected samples.

### Study Outcomes

Study primary outcomes included participants’ attitudes about using the home-based self-testing cervical cancer screening method, their perceived risk of HPV infection, related information on health and healthcare seeking behaviors, and the analyzability of self-collected cervical samples.

### Sample Size

We aimed to recruit a convenience sample of 30–60 participants that was expected to be sufficient to demonstrate implementation feasibility [37].

### Research Ethics

The study was approved by the study team’s university Institutional Review Board. At the phone screening call with the study coordinator, the women completed verbal informed consent to participate our study.

## Results

### Participant Recruitment

Overall, 224 women were contacted over a 5-month period from 2021 to 2022. Almost half (102/224, 45.5%) of the contacted women were ineligible for our study because they had a recent Pap test. Some women (32/224, 14.3%) were not eligible because they had their cervix completely removed. Additionally, twenty women (8.9%) declined to participate as they would not receive the HPV self-testing result because our study team’s research and administration capacity had not been ready for this analysis (Table 1). This decline led to a change in the protocol, once we were able to administer the analyses, that would allow us to provide the self-testing analysis results and offer information for follow-up referral to the participants. A total of 58 eligible and consented women were enrolled, of whom 35 (60.3%) returned both completed questionnaires and test kits and who were therefore considered for our analysis. Most of our participants (86.2%) were recruited by the community health workers.

### Participant Characteristics

Almost half (48.6%, 17/35) the participants were aged 55–64, with 32 (91.4%) African American. Most (71.4%) reported an annual income of < $40,000 (~ 150% federal poverty line for a family of four in the state) [38], almost half 16 (45.7%) relied on public insurance and another nine (25.7%) reported having no insurance. Most (68.6%) reported daily use of internet and had a smartphone (80%). The educational levels varied widely, with 15 (42.9%) being high school graduates, 10 (28.6%) having some college experience, and six (17.1%) being college graduates. Approximately every second participant reported experiencing their first sexual activity at ages 16–18 years (51.4%), having had more than 5 lifetime sexual partners (48.6%), and being comfortable with using tampons (57.1%) (Table 2).

### HPV Knowledge and Healthcare Seeking Behavior

The majority (~ 90%) reported not having ever been diagnosed with either HPV infection, cervical disease, oral or anal cancer. The percentages of participants who had a wrong or no answer to the questions on “HPV causes herpes.”, “HPV infection is rare.” and “HPV causes cervical cancer” were 77.2%, 37.2%, and 40%, respectively.

Most participants (60%) reported a “lot of trust with doctors” while ten (28.6%) cited “a little trust with doctors”. Asking about the healthcare experience in 2019 (to avoid possible nuances from the start of the COVID-19 pandemic in 2020), three of every four women reported having at least two doctor visits; and overall, all women had at least one doctor visit except one woman refused to answer (Table 3).

### Experience with HPV Self-Collection

When asked about the HPV self-test kit, 21 (60%) communicated positive feelings, with 15 (42.9%) reporting it to be easy to use and 12 (34.3%) reporting having a sense of privacy. In addition, 20 (57.1%) strongly believed that the test was safe, and 27 (77.1%) had no issues with the instructions. Almost everyone (94.3%) was comfortable with the method of delivery via mail, 42.9% reported a little physical discomfort 31.4% reported a desire for assistance with performing the self-testing, and 34.2% reported concern about confidentiality. When asked to compare their experience of receiving cervical cancer screening in the clinic, 29 (82.9%) participants preferred the home test, while four (11.4%) were indifferent between the two choices (Table 4).

### Additional Solicited Feedback from the Participants

A total of 27 participants responded to the question about their willingness to pay for this HPV self-testing as a cervical cancer screening test. The mean and median amount that the participants were willing to pay for this HPV self-testing were both about $20 (max: $50), but four women assigned $0, indicating an unwillingness to pay.

One participant suggested having a class or video to demonstrate use of the kit, one wanted the Evalyn^®^ instructional material to be more inclusive of African American women, and two expressed the interest in additional information to cope with the challenge of using the brush when experiencing “dryness” and additional information for clearer instructions.

The study coordinator and the participants communicated by telephone and phone text messages. Some participants preferred not to receive a cervical cancer-related text but ones with non-sensitive health content. One participant declined both to answer the questionnaire and to proceed with the self-test because she was afraid that she might “mess her stuff [daily life] up” and “would end up in the hospital”. Four participants who indicated they would send back the completed materials never did so.

### Administration and Laboratory Procedure for Self-Collected HPV Testing Samples

It took on average 20.5 days to receive the samples back (along with the completed paper questionnaires) from the day they were sent out (min–max: 8–71, median-18 days). The self-collected samples arrived at our study office in small numbers: 1–5 samples per arrival while the lab proceeded the analysis for dozens of samples per time. For economic reasons, we ran the samples into two batches. Thus, on average, 35 days were added to the waiting time between the specimen collection and the analysis. Overall, it took an average of 55.5 days from the sample collection time to the analysis. During the transit of the samples, the outdoor temperature was, on average, 62.7°F (min–max: 51.5°F–72.1°F, median-62.6°F) and there were no days with temperature < 39.2°F or > 98.6°F [39].

**Fig. 2:**
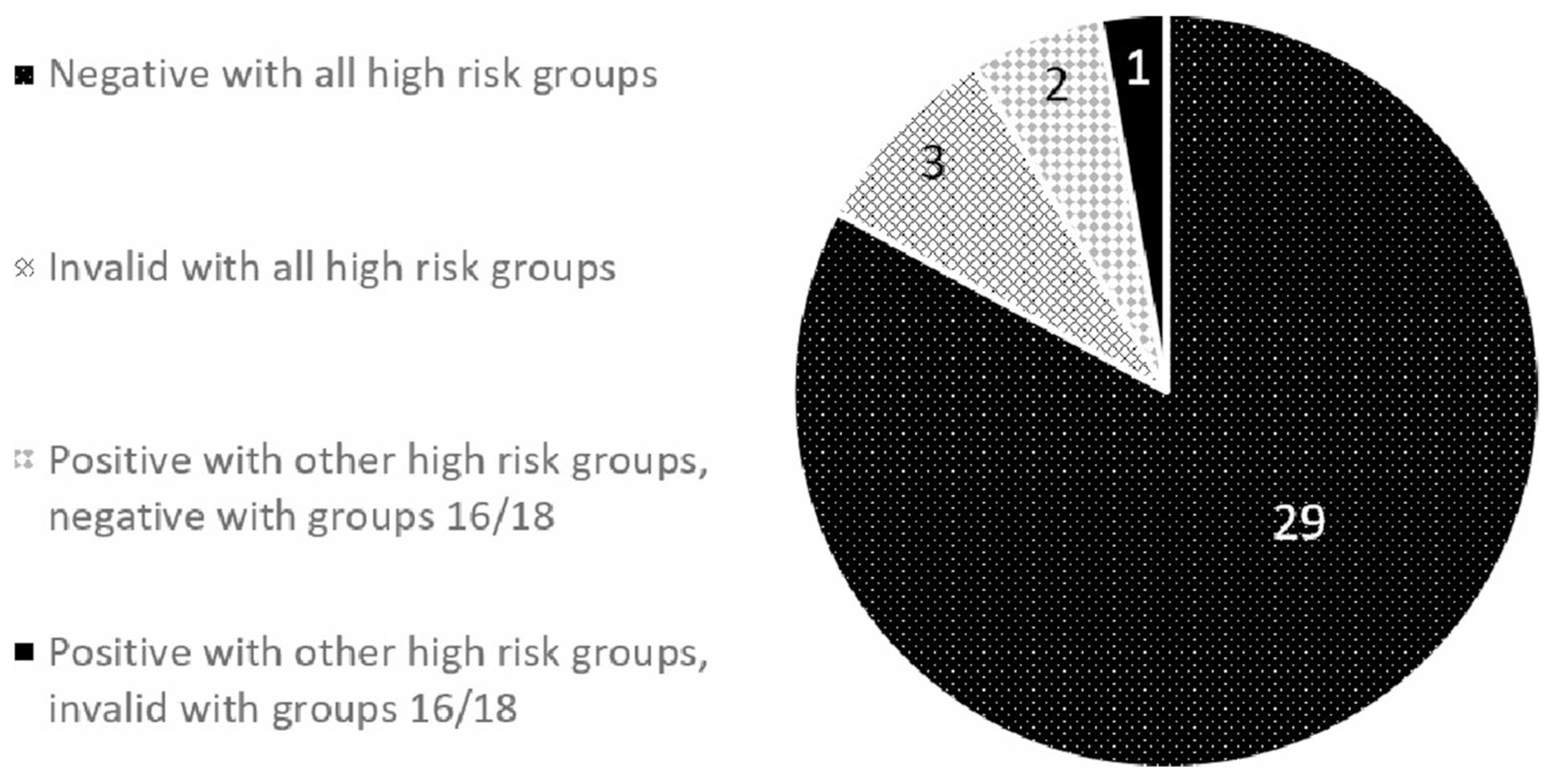
HPV testing results (N = 35). Decomposition of the HPV testing results among the recruited participants

More than 90% (32/35, 91.4%) of the received HPV self-testing samples were valid, producing 29 negative results against both high-risk-16 and 18 types and other high-risk groups, two positive results with high-risk-16 and 18 types but negative with other high-risk groups, and 1 positive with high-risk-16 and 18 types but invalid with other high-risk groups. Three samples were invalid showing as if no biosample was available on the brush for testing (Fig. 2).

## Discussion

This pilot study has yielded useful information about the feasibility and acceptability of mailed at-home HPV self-sampling test kits for cervical cancer screening among predominantly low-income African American women in Alabama. Since HPV is mainly transmitted through sex [5], and almost half of the participants reported more than five lifetime sexual partners, our study participants have the profile of women at higher risk of developing cervical cancer and most in need of improved screening service options [2].

In the United States, over 80% of U.S. females are infected with HPV at least once during their lifetime [40] and age-based prevalence of HPV infection among 18–60 year-old women ranges from 20 to 30% [41]. None of our participants reported to have ever been diagnosed with HPV previously. Overall, study participants exhibited poor HPV knowledge and low cervical health literacy [42, 43], a persistent impediment to cervical cancer screening uptake [9]. In addition, African American women cite trust in their healthcare providers and in the healthcare system as strong factors underpinning their cervical cancer screening uptake [44, 45]. Since a quarter of participants in this study reported having little trust, this indicates a mechanism for potentially improving cervical cancer screening uptake [44, 45] that future research and public health programs would need to address.

Most study participants (77.1%) considered the test kit easy to use, but some were unsure if they used it correctly (11.4%), and some needed extra help with the instructions (25.7%). Other studies [19] have also reported ease of use to be important to women. Overall, our findings from the survey and the participants’ feedback suggest a need for greater clarity in the instruction manual, on the instructional video by the Rover^®^ manufacturer [46], and on language inclusivity. Further, the study adds to the literature with information on the extent of technology usage in this specific population. A high proportion of our participants reported using the Internet daily and own a smartphone, indicating the potential for integrating mHealth in delivering the cervical cancer screening uptake as well as health education activities [47].

Almost all the women (94.3%) were comfortable with receiving the test-kit delivery by mail. The convenience of mail-delivered HPV test kits has been considered as a solution to address structural barriers to access to care (including distrust of the medical system and financial barriers) and individual-level barriers to access care (including childcare, transportation) [48]. A randomized trial in Denmark found women were more likely to participate in mailed at-home testing than standard testing [15]. In the US, success in mailed at-home HPV testing for improving cervical cancer screening uptake has been seen in places where electronic health records are used to identify underscreened women, as also found in large managed health care systems like Kaiser Permanente [49] and safety net healthcare settings. [23].

There remains much uncertainty regarding how best to bridge women in need of cervical cancer screening with the at-home self-testing service. The electronic health records strategy used in other areas may not be applicable in Alabama. Previous studies in the southern US have suggested the potential for mailed cervical cancer screening tests to be used in federally qualified health centers [50], in a randomized clinical trial settings with multiple recruitment strategies in North Carolina [51], in a community health worker-led recruitment in south Florida [52], and in door-to-door recruitment by community health workers in rural Mississippi [53]. Our study focused on underscreened women recruited from community settings and recruited by community health workers [26]. We continue to emphasize the role of community health workers in cervical cancer screening interventions including home-based HPV self-testing programs because these workers can be a bridge to women in need of screening with the service. Trained community health workers are frontline public health workers who have recently been endorsed and certified by the Alabama Department of Public Health via HB615 bill [54]. The community health worker model holds strong promise for improving the effectiveness and cost-effectiveness of home-based cervical cancer screening in the community setting in Alabama and other Deep South states which are the most disproportionately affected by cervical cancer [1].

Connor et al. reported a timeframe of 12 weeks from collection to analysis and the temperature range between 39.2°F and 98.6°F to warrant ample sample integrity and support laboratory analysis for the HPV self-collected samples [39]. In our study, samples were received for testing 50–60 days on average after mailing out the kits. This fits well with the 12-week timeframe, indicating the feasibility of administering this mailed self-testing service at scale. Our study sample collection occurred in cool weather months which is an important consideration given that the temperature in Alabama can exceed 100°F in the height of summer [55].

This study has several limitations. This was a pilot study whose small sample was identified by convenience sampling and recruitment was disrupted by the early phase of the COVID-19 pandemic, prolonging the recruitment period. However, all study procedures were carefully followed. Three self-collected cervical samples were invalid, which suggests the need to closely monitor the study procedures and to further validate such self-collection.

The FDA approval of the self-testing HPV modality offers scope for improving cervical cancer screening through enhancing deployment of this self-testing method. This pilot study contributes new evidence to help bridge the “know-do” gaps [56] for developing future studies and interventions using HPV self-testing. This would also help accomplish the goals of Operation WIPE OUT [27], the current statewide initiative to eliminate cervical cancer in Alabama, and programs in other states to end this preventable scourge in our lifetime.

## Figures and Tables

**Table 1:** Recruitment information for consented participants and the women who declined to participate

	CHW1	CHW2	CHW3	Study coordinator	Total
Contacted	100	80	32	12	224
Participated	23	17	10	8	58
Refused/Was ineligible to participate					
Had total hysterectomy	10	20	0	2	32
Had a recent Pap testing	62	28	11	1	102
Didn’t participate for no results*	5	15	0	0	20
Was not African American or not living in rural areas	0	0	0	1	1
Was not in age range	0	0	3	0	3
Refused with no reason given	0	0	8	0	8

CHW, community health worker

* We had not planned for returning the HPV testing results at first; then, we changed our protocol based on multiple participants’ requests having access to their results

**Table 2:** Participant characteristics (N = 35)

Characteristic	No. (%)
Age (years)	
30–44	6 (17.1%)
45–54	12 (34.3%)
55–64	17 (48.6%)
Race	
Black or African American	32 (91.4%)
White	3 (8.6%)
Marital Status	
Single/Never Married	12 (34.3%)
Divorced	11 (31.4%)
Married/Cohabiting	9 (25.7%)
Widowed	2 (5.7%)
Refused	1 (2.9%)
Education	
Some High School	2 (5.7%)
GED or High School Graduate	15 (42.9%)
Some College	10 (28.6%)
College Graduate	6 (17.1%)
Refused	2 (5.7%)
Income	
< $40,000	25 (71.4%)
$40,000—$79,999	5 (14.3%)
$80,000 +	3 (8.6%)
Refused	2 (5.7%)
Insurance Type	
None	9 (25.7%)
Public	16 (45.7%)
Private	7 (20%)
Refused	3 (8.6%)
Smoking	
Yes	4 (11.4%)
No	31 (88.6%)
Internet	
Daily	24 (68.6%)
≤ Weekly	6 (17.1%)
Don’t Know	1 (2.9%)
Refused	4 (11.4%)
Smartphone	
Yes	28 (80%)
No	4 (11.4%)
Refused	3 (8.6%)
Help with Medical and Pharmacy Instructions	
Yes	9 (25.7%)
No	26 (74.3%)
Parity	
0	8 (22.9%)
1	6 (17.1%)
2	9 (25.7%)
3+	12 (34.3%)
Using Tampon	
Comfortable	20 (57.1%)
Not Comfortable	5 (14.3%)
Never Used	8 (22.9%)
Don’t Know	1 (2.9%)
Refused	1 (2.9%)
Sexual Debut	
12–15	6 (17.1%)
16–18	18 (51.4%)
19+	3 (8.6%)
Don’t Know	4 (11.3%)
Never Had Sex	1 (2.9%)
Refused	3 (8.6%)
Sex Partners	
< 5	13 (37.1%)
6–10	9 (25.7%)
11–49	6 (17.1%)
50 +	2 (5.7%)
Refused	5 (14.3%)

**Table 3:** Health and healthcare seeking behaviors and HPV knowledge (N = 35)

Characteristic	No. (%)
Hysterectomy	
Yes	13 (37.1%)
No	22 (62.9%)
Diagnosed with HPV	
Yes	1 (2.9%)
No	32 (91.4%)
Don’t Know	1 (2.9%)
Refused	1 (2.9%)
Diagnosed with Cervical Disease	
No	33 (94.3%)
Don’t Know	1 (2.9%)
Refused	1 (2.9%)
Ever Tested Positive for HPV	
No	31 (88.6%)
Don’t Know	3 (8.6%)
Refused	1 (2.9%)
Diagnosed with Oral/Anal Cancer	
No	31 (88.6%)
Don’t Know	3 (8.6%)
Refused	1 (2.9%)
Doctor Visits (2020)	
0–2	10 (28.6%)
> 2	23 (65.7%)
Refused	2 (5.7%)
Doctor Visits (2019)	
0–2	8 (22.9%)
> 2	26 (74.3%)
Refused	1 (2.9%)
Trust in Doctors	
A little	10 (28.6%)
A lot	21 (60%)
Don’t Know	2 (5.7%)
Refused	2 (5.7%)
HPV Infection is Rare	
Yes	3 (8.6%)
No	22 (62.9%)
Don’t Know	10 (28.6%)
HPV Causes Cervical Cancer	
Yes	21 (60%)
No	3 (8.6%)
Don’t Know	11 (31.4%)
HPV Causes Genital Warts	
Yes	13 (37.1%)
No	5 (14.3%)
Don’t Know	17 (48.6%)
HPV Causes Herpes	
Yes	10 (28.6%)
No	8 (22.9%)
Don’t Know	17 (48.6%)
HPV is Contracted Through Sexual Contact	
Yes	17 (48.6%)
No	4 (11.4%)
Don’t Know	14 (40%)
HPV is Curable	
Yes	11 (31.4%)
No	5 (14.3%)
Don’t Know	19 (54.3%)

**Table 4:** Acceptability of the self-test kit and problems with use (N=35)

Characteristic	No. (%)
Acceptability	
Overall Thoughts	
Positive	21 (60%)
Neutral	10 (28.6%)
Don’t Know	4 (11.4%)
Positive Feedback*	
Ease of Use	15 (42.9%)
Privacy	12 (34.3%)
Comfortability	3 (8.6%)
Other	4 (11.4%)
Comfortable Receiving in Mail	
Yes	33 (94.3%)
No	1 (2.9%)
Don’t Know	1 (2.9%)
Confidence in Correct Application	
Strongly Agree	14 (40.0%)
Somewhat Agree	15 (42.9%)
Somewhat Disagree	4 (11.4%)
Don’t Know	1 (2.9%)
Refused	1 (2.9%)
Think Test is Safe	
Strongly Agree	20 (57.1%)
Somewhat Agree	11 (31.4%)
Don’t Know	3 (8.6%)
Refused	1 (2.9%)
How Difficult to Understand Instructions	
Not Difficult	27 (77.1%)
Somewhat Difficult	6 (17.1%)
Refused	2 (5.7%)
Preference of Cervical Cancer Screening: Home vs. Clinic	
Home	29 (82.9%)
Clinic	1 (2.8%)
Same	4 (11.4%)
Refused	1 (2.8%)
Negative Feedback*	
Physical Discomfort	4 (11.4%)
Unsure if Correct Application	4 (11.4%)
Standing Use	1 (2.9%)
Self-Use	1 (2.9%)
Physical Discomfort	
A Little	15 (42.9%)
None at All	18 (51.4%)
Refused	2 (5.7%)
Injury	
Yes	3 (8.6%)
No	30 (85.7%)
Don’t Know	1 (2.9%)
Refused	1 (2.9%)
Wanted Assistance	
Agree	11 (31.4%)
Disagree	20 (57.1%)
Don’t Know	1 (2.9%)
Refused	3 (8.6%)
Hard to Find Time	
Agree	8 (22.9%)
Disagree	24 (68.6%)
Don’t Know	1 (2.9%)
Refused	2 (5.7%)
No Privacy	
Agree	4 (11.4%)
Disagree	28 (80%)
Don’t Know	2 (5.7%)
Refused	1 (2.9%)
Friends or Family Discouraged	
Yes	2 (5.7%)
No	1 (2.9%)
Concerned about Confidentiality	
Agree	12 (34.2%)
Strongly Disagree	18 (51.4%)
Don’t Know	4 (11.4%)
Refused	1 (2.9%)

* The percentages do not add to 100% due to the lack of answers from some participants
